# When Threat Comes from Within: First Evidence that Anticipatory Emotion Impairs Working Memory

**DOI:** 10.1007/s42761-026-00363-y

**Published:** 2026-03-09

**Authors:** Hippolyte Fournier, Lou Pardon Tommasini, Aurélie Walter, Gaën Plancher

**Affiliations:** 1 Laboratoire d’Étude des Mécanismes Cognitifs, Université Lyon 2, Lyon, France; 2 Institute of Psychology, University of Lausanne, Bâtiment Géopolis, Quartier UNIL-Mouline, Lausanne, CH-1015 Switzerland; 3 Institut Universitaire de France, Lyon, France

**Keywords:** Anticipatory threat, Working memory, Emotion, Appraisal, Anxiety

## Abstract

Although anticipating threat is known to capture attentional resources, its direct impact on working memory (WM), a core cognitive function supporting everyday activities, has yet to be empirically examined. Given the close dependence of WM on attentional resources, it is therefore reasonable to hypothesize that anticipating threat disrupts WM performance. Moreover, since threat-related attentional bias increases with anxiety, WM disruption caused by anticipatory threat may likewise intensify with anxiety. In Experiment 1, we tested whether anticipating threat captures attentional resources and impairs WM, and whether these effects vary with anxiety. Experiment 2 replicated and extended this design under higher task demands. Participants performed a gamified complex-span task in which each trial occurred in a virtual room associated with the potential appearance of a proximal threat (a sudden screamer), a distal threat (loss of accumulated points), or a neutral condition. Importantly, no threat ever appeared, isolating purely anticipatory processes. Attentional capture was indexed by parity-judgment accuracy, and WM by serial recall. Across both experiments, threat anticipation produced attentional capture and impaired WM. In Experiment 1, attentional capture decreased as anxiety increased, whereas this compensatory pattern disappeared under higher task load in Experiment 2. In contrast, WM impairment increased with cognitive anxiety, particularly under distal threat. These findings provide the first empirical evidence that anticipating threat impairs WM, in line with recent models of emotion, anxiety, and WM.

**Supplementary Information:**

The online version contains supplementary material available at 10.1007/s42761-026-00363-y.

Over recent decades, the anticipatory nature of brain function has gained momentum in the literature, portraying the brain as a system that continuously generates predictions from which perception and action emerge (Friston et al., 2017; Hohwy, 2013). Within this framework, emotional responses are not merely reactive but arise proactively, preparing the organism for possible outcomes before they materialize (Seth, 2013; Seth & Friston, 2016). Anticipatory emotion thus reflects a core computational principle, relying on internally generated representations that enable the organism to prepare for potentially relevant future states. Threat provides a particularly illustrative example. In the temporal unfolding of threatening interactions, the pre-encounter phase, when no threat is present yet the likelihood of encountering one is high, corresponds to threat anticipation (Mobbs et al., 2020). This phase involves assessing potential negative outcomes and determining how to respond to them. Appraisal theories of emotion help clarify the mechanisms underpinning this anticipatory phase. They place evaluative processes at the heart of emotion elicitation. According to these accounts, emotion arises from an internal appraisal of the significance of an event relative to an organism’s major concerns (Grandjean et al., 2008; Sander, 2013). Threat is appraised based on its relevance, expected consequences, and one’s coping potential (Scherer, 2009; Smith & Kirby, 2009). This evaluative process orchestrates coordinated physiological, cognitive, and behavioral adjustments that prepare the organism for an adaptive response. A central implication is that when a situation is appraised as potentially threatening, attentional resources are preferentially allocated to that threat (Sladky et al., 2024; Vuilleumier, 2005). Empirically, numerous studies showed that the presentation of negative or threat-related stimuli automatically biases attention. This has been demonstrated for proximal threats, whose negative consequences are immediate or temporally close (e.g., a sudden aversive noise; Vogt et al., 2013; Fournier and Koenig, 2024), as well as distal threats, whose consequences are more abstract and temporally removed, such as cues signaling potential loss of points in a goal-oriented context (Müller et al., 2016; Wentura et al., 2014). Importantly, these attentional effects show substantial inter-individual variability. Indeed, anxious individuals typically display stronger threat-related attentional biases (Bar-Haim et al., 2007; Fournier et al., 2025), consistent with the tendency of anxiety to overestimate negative outcomes (Eysenck et al., 2007). While this literature has primarily examined attention to presented threat, several studies also showed that merely anticipating negative events captures attentional resources, diverting attention away from ongoing task demands during performance (Kleinsorge, 2007, 2009). These findings indicate that threat, and even its anticipation in the absence of sensory input, can bias attention.

What remains unknown, however, is whether anticipatory threat also disrupts other cognitive functions, such as working memory (WM). WM is defined as the temporary maintenance and manipulation of information (Baddeley & Hitch, 1974). This question is central because WM underlies many higher-order processes essential for everyday activities, including language comprehension, reasoning, and spatial cognition. WM is particularly well suited for addressing this issue given its close dependence on attentional resources. According to the Time-Based Resource Sharing (TBRS) model, WM relies on the dynamic and time-constrained allocation of a limited attentional resource shared between maintaining memoranda and processing concurrent cognitive demands, creating inherent competition for this resource (Barrouillet et al., 2011; Plancher & Barrouillet, 2020). Memory traces begin to decay as soon as attention is diverted, and their preservation requires periodic attentional refreshing, a mechanism that briefly reinstates items in the focus of attention to keep them active (Camos & Barrouillet, 2014). Thus, any factor that reduces the availability of attentional resources, whether external or internally generated, should impair WM performance.

This prediction receives indirect support from two lines of work. First, presenting emotional stimuli during a concurrent task impairs WM performance, typically reducing recall accuracy in complex span paradigms (Chainay et al., 2023; Colliot et al., 2025; Garrison & Schmeichel, 2018; Plancher et al., 2019; Schweizer & Dalgleish, 2011, 2016). Threatening stimuli capture attention, thereby limiting the resources available to maintain memoranda. Second, the impact of internally generated representations on WM has long been theorized within WM models (Cowan, 2005). However, this hypothesis has so far been tested only in non-emotional contexts, using mental simulations or spontaneous internal distractions (Kiyonaga & Egner, 2016; Yu & Postle, 2021). Together, these findings suggest that anticipatory threat, much like other internal distractors, may impair WM by taxing the attentional resources needed to sustain active representations.

Taken together, this state of the art converges on a clear prediction. If anticipating a threat draws on limited attentional resources, and if WM depends on these same resources, then anticipatory threat should impair WM performance. Yet, to our knowledge, this prediction has never been directly tested. Individual differences further strengthen this rationale: threat-related capture is often amplified among anxious individuals (Bar-Haim et al., 2007; Fournier et al., 2025), and similar patterns have been observed for WM in individuals with higher cognitive anxiety (Angelidis et al., 2019). If anticipatory threat taxes attentional resources in a comparable way, its impact on WM should increase with cognitive anxiety.

Here, we developed the first experimental paradigm designed to formally test whether anticipatory threat impairs WM, and whether this effect is moderated by cognitive anxiety. These hypotheses were tested using two forms of anticipatory threat, a proximal threat (anticipation of a sudden screamer) and a distal threat (anticipation of losing accumulated points), both known to bias attention (Fournier & Koenig, 2024; Müller et al., 2016; Vogt et al., 2013; Wentura et al., 2014). These, along with a neutral condition, were embedded in a gamified complex-span task in which participants earned points by recalling letters interleaved with parity judgments while navigating the rooms of a virtual mansion. Before the task, participants were introduced to three hosts tied to room colors and described as potentially appearing at any moment. Critically, no host ever appeared during the task. The manipulation relied solely on anticipation, allowing us to isolate its effects without introducing any sensory threat stimuli.

Across two experiments differing in task difficulty, two predictions were tested: anticipatory threat should capture attentional resources and impair WM relative to a neutral condition, and these effects should increase with cognitive anxiety. Experiment 1 provided an initial test using five letters, each separated by two digit parity judgments. Experiment 2 then replicated and extended the design under higher cognitive demand by presenting six letters interleaved with three digits. Parity-judgment accuracy served as an index of attentional capture, and recall accuracy as a measure of WM performance, with the expectation that changes in the former would be reflected in the latter.

## Experiment 1

### Method

#### Participants

Forty-seven undergraduate students from Lyon 2 University participated in the experiment (mean age = 20.25 years, SD = 2.75; 18 men, 27 women, 2 identified as “other”). To ensure that participants were actively engaged in the concurrent parity task, we excluded from analysis those who scored below $70\%$ accuracy on this task, as done in previous studies using complex span paradigms (Camos et al., 2019; Labaronne et al., 2024, 2023). This led to the exclusion of 7 participants, resulting in a final sample of 40 participants included in the analysis.

The experiment was conducted in accordance with the principles of the Declaration of Helsinki. All participants provided written informed consent and were informed prior to the task that they might encounter potentially threatening stimuli. They were also reminded of their right to withdraw from the study at any time without penalty.

Sample size was determined based on previous studies using complex span tasks to examine the effect of the presentation of negative stimuli on working memory performance. These studies reported a mean effect size of $d = 0.54$ (Chainay et al., 2023; Colliot et al., 2025) and require at least 30 participants to obtain $90\%$ power at $\alpha$ = 0.05. Given the increased complexity of our design, featuring two threat conditions and an interaction with anxiety, we increased our target sample size to 40 participants. A post hoc power analysis based on the observed z-values for the expected fixed effects that reached significance indicated that all of them achieved a power of at least $90\%$.

#### Materials and Procedure

The task was programmed using OpenSesame 4.0 (Mathôt et al., 2012). Visual materials, including background images, room colors, and score displays, were created using Affinity Photo 2 (Serif Europe Ltd.), and animated transitions were designed in Microsoft PowerPoint. The facial images representing the hosts were generated using an artificial intelligence tool that produces royalty-free images for non-commercial use (https://starryai.com/). Participants wore headphones throughout the experiment, and immersive ambient soundtracks were played to reinforce the atmosphere of each room. These audio clips were drawn from the International Affective Digitalized Sounds, Expanded Version (IADS-E; Yang et al., 2018).

The experiment was designed to immerse participants in a gamified environment in which they completed a point-based task set in a virtual mansion. Participants were instructed that their goal was to score as many points as possible by recalling consonant letters in the correct order and accurately performing parity judgments. Each trial was said to take place in a different colored room, red, green, or blue, and participants were told that each room might be inhabited by a “host” who could appear at any time. The association between room color and host identity was randomized across participants. Sybille, the neutral host, was described as friendly and non-threatening; her footsteps were audible before entering the room to avoid startling participants. Vlad, the proximal threat host, was described as hostile and prone to appearing suddenly and screaming to frighten participants. Max, the distal threat host, was described as calm and non-violent but said to steal all accumulated points if he appeared. Each host was introduced with a specific musical cue matching their role: a neutral tone for Sybille, a suspenseful and threatening sound for Vlad, and a feedback tone commonly used in video games to signal point loss for Max. Importantly, although participants were told that hosts might appear at any moment, no host ever actually appeared during the task. Threat was therefore entirely anticipatory.

Before the experiment began, participants provided written informed consent and completed a brief sociodemographic questionnaire, followed by the Penn State Worry Questionnaire (PSWQ; Gosselin et al., 2001) to assess cognitive anxiety. They were then introduced to the game narrative and task instructions through an animated video that explained the context, objectives, and the potential role of each host. To familiarize participants with the context, each host was explicitly introduced in a short practice sequence. Participants completed one trial in each room (one per host), during which they were told that the corresponding host would appear. This exposure phase ensured that participants formed clear expectations about each room’s associated threat.

**Fig. 1:**
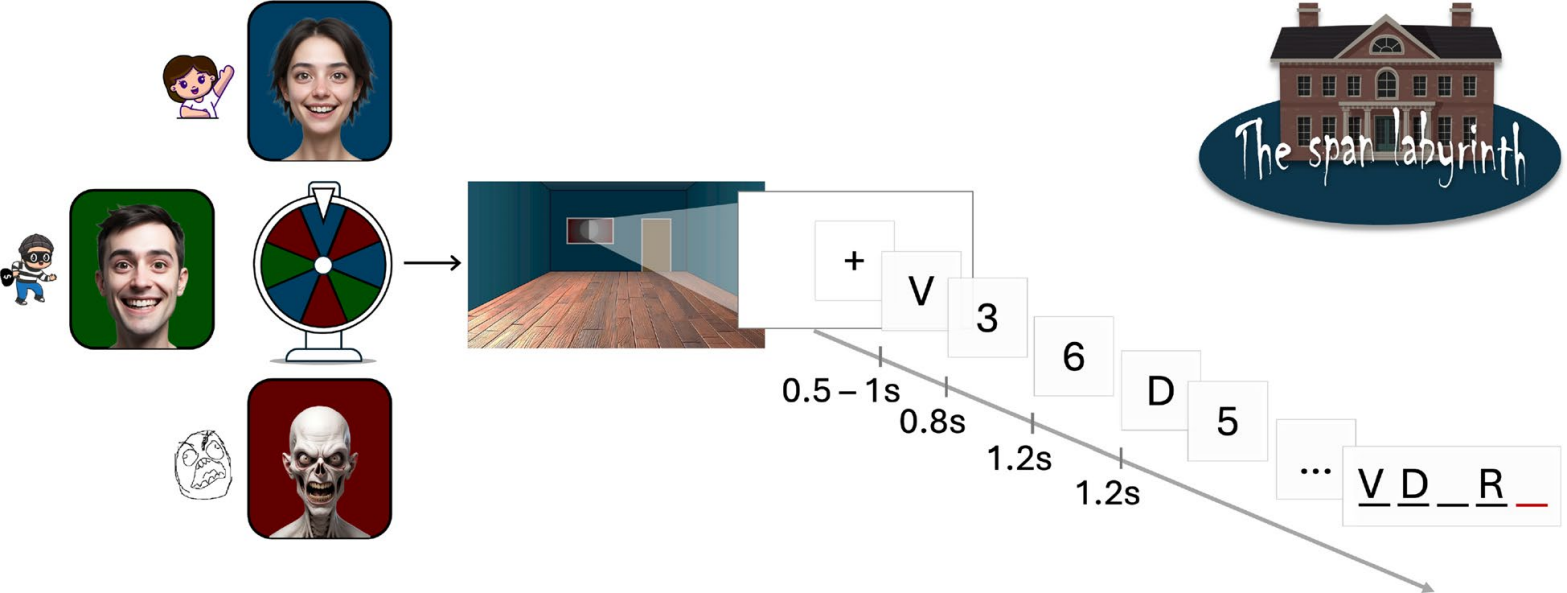
Illustration of the experimental design used in the Span Labyrinth task, a gamified version of a complex span paradigm. *Note.* In each trial, participants viewed a sequence of five consonant letters presented one at a time, each separated by two digits requiring a parity judgment (odd/even) via keyboard response. At the end of the sequence, participants were prompted to recall all five letters in order. The task was embedded in a point-based game context: Each correct letter recall (i.e., recalled in the correct position) earned 3 points, and each correct parity judgment earned 1 point. Trials took place in different virtual rooms of a mansion, each associated with a distinct threat condition, indicated by room color and a narrative involving a potential “host” who could appear at any time (though no host ever appeared). The room-to-condition assignment (blue, green, red) was randomized for each participant. In the neutral condition (e.g., blue rooms), Sybille, the neutral host, was said to have no effect on the game. In the distal threat condition (e.g., green rooms), Max, the distal threat host, was said to be able to appear at any moment and instantly erase all accumulated points. In the proximal threat condition (e.g., red rooms), Vlad, the proximal threat host, was described as potentially appearing at any moment to startle the participant

After the introduction, participants completed a three-phase practice session. In the first phase, they performed 54 untimed trials of the parity task alone. In the second phase, they practiced three trials of the recall task in isolation. The third phase consisted of three full trials of the complex span task, combining both letter recall and parity judgment components (Fig. 1).

The main experimental task included 27 trials of the complex span task. Each trial began with a fixation cross displayed for 500 to 1,000 ms, followed by five consonant letters presented sequentially for 800 ms each. Between each letter, participants performed a parity judgment task involving two digits. Each digit was presented for 1,000 ms with a 200 ms inter-stimulus interval, and participants judged whether the digit was odd or even using the “s” (odd) or “m” (even) key on an AZERTY keyboard. Digits disappeared upon keypress, and timing was adjusted to maintain a consistent 1,200 ms interval between stimuli. After each trial, participants were prompted to recall and type the five letters in the correct order using five response boxes, with no time limit. The response was submitted by pressing the Enter key. Each correctly recalled letter in its correct serial position earned 3 points, and each correct parity judgment earned 1 point. Participants were reminded of these scoring rules at the end of the instruction phase. They were also reminded that each trial would take place in a colored room associated with a specific host, and that the host might appear unexpectedly during the trial. After completing the structured introduction, the experimenter repeated the instructions, turned off the lights in the testing booth, and left the room to increase immersion. A short break was offered at the midpoint of the task, during which participants were reminded of their current score. The second half of the task resumed under the same conditions.

At the end of the experiment, participants completed a short computerized debriefing questionnaire using continuous sliders ranging from 0 to 1 (step size = 0.01), operated with the mouse. They indicated the extent to which they had wanted to achieve the highest possible score (from Not at all to Very much). They also rated how pleasant or unpleasant they would have found the appearance of each host (from Unpleasant to Pleasant, with Neutral as the midpoint), and how much they had dreaded each host’s potential appearance (from Not at all to Very much). Finally, they estimated how likely they thought each host was to appear during the first and second halves of the game (i.e., before and after the mid-task break), using the same response scale.

#### Statistical Analyses

Statistical analyses were conducted in R (version 4.3.1). We first examined questionnaire responses to evaluate the effectiveness of the anticipatory threat induction. Main analyses focused on parity-judgment accuracy (correct vs. incorrect odd/even classification) and recall accuracy (letter recalled in the correct serial position vs. not), used respectively as indices of attentional capture and WM performance. Both measures were analyzed separately using binomial Generalized Linear Mixed-Effects Models (GLMMs). For each task, the fixed-effects structure included Threat condition (Neutral, Proximal threat, Distal threat), PSWQ scores (continuous measure of cognitive anxiety; Gosselin et al., 2001), and their interaction. PSWQ scores were computed as the sum of the 16 items (range: 16–80). Internal consistency in our sample was excellent (Cronbach’s $\alpha$ = 0.92; *M* = 52.67, *SD* = 9.02), slightly below the French validation sample (M = 62.55, SD = 9.06).

Random-effects structures of the GLMMS were identified following the recommendations of Barr et al. (2013). For each task, the maximal structure justified by the design and retained the version that best accounted for the data was selected. It included random intercepts for participants and random slopes for all within-participant variables: Threat condition, trial number (1–27), indexing task progression, and item position (letter or digit), indexing the position of each item within a trial. Item position was recoded to capture the primacy/recency structure based on a data-driven analysis (see Supplementary Material). All possible random-effects substructures were then compared using the Corrected Akaike Information Criterion (AICc; Burnham and Anderson, 2004), and the model that converged and minimized AICc was retained. This procedure was repeated independently for each task.

Hypothesis tests were conducted using the retained random-effects structure. The effect of Threat condition was evaluated by comparing a model with PSWQ only to a model including both PSWQ and Threat condition. The Threat condition $\times$ PSWQ interaction was tested by comparing a model including their main effects to the full model, additionally including their interaction. Additionally, an exploratory analysis examined whether these effects varied across the progression of the task by adding a Partition factor (first block: first 14 trials; second block: last 13 trials) to the models. Effects were evaluated using likelihood-ratio tests and changes in AICc (with $\Delta$AICc $> 2$ interpreted as evidence supporting the effect).

When an effect was significant, planned contrasts were computed using the full model. For the main effect of Threat condition, the Proximal and Distal threat conditions were each compared to the Neutral condition. For the Threat condition $\times$ PSWQ interaction, the change in threat–neutral differences along PSWQ was examined. Contrasts were tested using Wald z-tests, and standardized log-odds estimates are reported together with their standard errors and 95% Confidence Intervals (95% CI), allowing for straightforward interpretation of effect sizes. In cases involving multiple comparisons, Tukey-corrected p-values were reported. All continuous predictors (fixed and random) were z-scored. Finally, a Pearson correlation was computed between parity-judgment accuracy and recall accuracy to examine whether the degree of attentional capture was related to working-memory performance.

### Results

#### Questionnaire

To validate the effectiveness of the narrative manipulation used to induce threat anticipation, we analyzed participants’ post-task questionnaire responses. Full details are provided in the Supplementary Material (Figures S2). Participants reported strong engagement with the gamified task. As expected, both the proximal-threat and distal-threat hosts were rated more unpleasant than the neutral host, with no significant difference observed between the two threat conditions. Moreover, fear of appearance was higher for the two threat-related hosts than for the neutral host. Participants also reported anticipating the hosts’ appearance throughout the task. Overall, the data support the plausibility and internalization of the threat manipulation.

#### Parity Judgment

Mean parity-judgment accuracy was high (*M* = 0.85, *SD* = 0.07). The best-fitting random-effects structure included random intercepts for participants, together with uncorrelated random slopes for trial number, both varying within participants (see Supplementary Material for details). Fixed effects were then tested using this structure ($R^2_{\text {marginal}} = 0.01$, $R^2_{\text {conditional}} = 0.15$).

Removing Threat condition did not improve model fit, $\chi ^2(2) = 4.44$, $p = 0.11$, $\Delta \textrm{AICc} = 0.04$, indicating no main effect of Threat condition. An exploratory analysis (reported in Supplementary Material) confirmed that this absence of effect did not vary across the progression of the task.

**Fig. 2:**
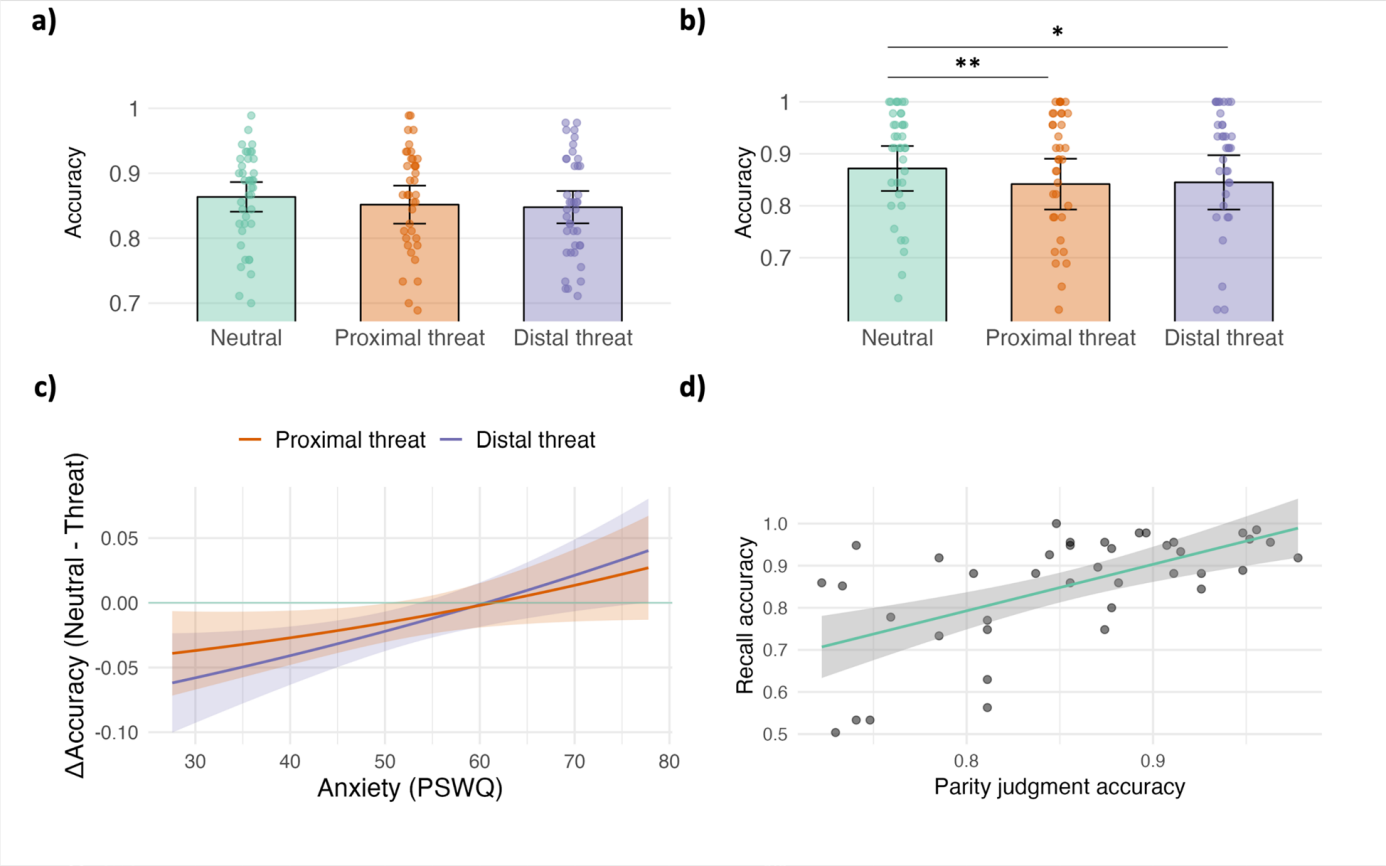
Illustration of accuracy patterns as a function of threat conditions and PSWQ scores in Experiment 1. **a**) Mean parity-judgment accuracy across threat conditions. **b**) Mean recall accuracy across threat conditions. **c**) Effect of PSWQ scores on the change in parity-judgment accuracy between threat and neutral conditions. **d**) Correlation between parity-judgment and recall accuracy. *Note.* For parity judgments, accuracy reflects the proportion of digits correctly classified. For recall, accuracy reflects the proportion of letters recalled in the correct serial position. In panels a) and b), vertical lines represent 95% confidence intervals; * $p < 0.05$, ** $p < 0.01$. In panel c), shaded areas represent 95% confidence intervals

By contrast, removing the Threat condition $\times$ PSWQ interaction significantly worsened model fit, $\chi ^2(2) = 11.27$, $p = 0.004$, $\Delta \textrm{AICc} = 7.30$, indicating a significant interaction. Planned contrasts showed that threat-related interference decreased as PSWQ increased, both for the Proximal threat ($\beta = 0.17$, *SE* = 0.07, 95% CI [0.02, 0.31], $z = 2.22$, $p = 0.027$) and the Distal threat ($\beta = 0.25$, *SE* = 0.07, 95% CI [0.10, 0.39], $z = 3.33$, $p < 0.001$). These effects are illustrated in Fig. 2a and c.

#### Recall

Mean recall accuracy was high (*M* = 0.85, *SD* = 0.13). The best-fitting random-effects structure included random intercepts for participants and uncorrelated random slopes for trial number and letter position, both varying within participants (see Supplementary Material). Fixed effects were then tested using this structure ($R^2_{\text {marginal}} = 0.01$, $R^2_{\text {conditional}} = 0.33$).

Removing Threat condition significantly worsened model fit, $\chi ^2(2) = 8.76$, $p = 0.013$, $\Delta \textrm{AICc} = 4.70$, indicating a significant main effect. Planned contrasts showed lower recall accuracy in both Proximal threat ($\beta = -0.29$, *SE* = 0.11, 95% CI [–0.49, –0.08], $z = 2.78$, $p = 0.005$) and Distal threat conditions compared to neutral condition ($\beta = -0.24$, *SE* = 0.11, 95% CI [–0.44, –0.03], $z = 2.25$, $p = 0.025$). Exploratory analyses confirmed that this threat effect was stable across task progression (see Supplementary material for details). Removing the Threat condition $\times$ PSWQ interaction also significantly worsened model fit, $\chi ^2(2) = 6.99$, $p = 0.030$, $\Delta \textrm{AICc} = 3.00$, but planned contrasts did not yield significant simple effects for Proximal threat $\times$ PSWQ ($\beta = 0.13$, *SE* = 0.11, 95% CI [–0.09, 0.34], $z = 1.13$, $p = 0.26$) nor Distal threat $\times$ PSWQ ($\beta = -0.17$, *SE* = 0.12, 95% CI [–0.40, 0.06], $z = -1.46$, $p = 0.14$). The Threat condition main effect is shown in Fig. 2b.

A subsequent analysis examined the association between performance on the two tasks. Parity-judgment accuracy and recall accuracy were positively correlated ($r = 0.60$, 95% CI [0.35, 0.76], $p < 0.001$), as shown in Fig. 2d.

Across both tasks, anticipatory threat impaired performance, but in different ways for attentional and WM processes. Parity-judgment accuracy showed no overall threat effect but revealed a clear Threat condition $\times$ PSWQ interaction: threat-related interference decreased as cognitive anxiety increased. Recall accuracy showed a robust detrimental effect of threat that was not moderated by PSWQ, and a modest Threat $\times$ PSWQ interaction that did not yield significant simple effects. Finally, the two tasks were positively correlated, indicating that individuals showing stronger attentional interference also tended to show greater WM disruption.

This progressive reduction in threat-related interference with increasing anxiety is consistent with the well-documented “threat-inhibition” pattern, interpreted as a regulatory strategy aimed at reducing anxiety (Iacoviello et al., 2014; Naim et al., 2015; Zvielli et al., 2014). Prior work suggests that this regulatory mechanism becomes difficult to maintain under increased cognitive load (Angelidis et al., 2019; Shi et al., 2019). Experiment 2 therefore sought to replicate and extend these findings by increasing task difficulty, with the prediction that higher cognitive demands would limit strategic control, amplify WM disruption, and strengthen its association with cognitive anxiety.

## Experiment 2

### Method

#### Participants

Forty-five undergraduate students from Lyon 2 University took part in Experiment 2 (mean age = 20.55 years, SD = 1.79; 10 men, 35 women). To ensure engagement in the parity task, participants with less than $70\%$ accuracy were excluded from the analysis (n = 5), following the same criteria as in Experiment 1. This resulted in a final sample of 40 participants included in the analyses.

As in Experiment 1, the study was conducted in accordance with the Declaration of Helsinki. All participants provided written informed consent and were informed of the potential presence of threatening stimuli, as well as their right to withdraw at any point. Because the statistical analyses followed the same approach as in Experiment 1, and the design complexity was comparable, we targeted the same sample size of 40 participants to ensure sufficient power. A post hoc power analysis based on the observed z-values for the expected fixed effects that reached significance indicated that all of them achieved a power of at least $93\%$.

#### Materials and Procedure

The materials and procedure were identical to those used in Experiment 1, except that each trial involved the sequential presentation of six consonant letters instead of five, each separated by three parity digits instead of two. This increase in task difficulty was designed to reduce participants’ ability to maintain control over performance, thereby amplifying potential effects of threat and cognitive anxiety on working memory and attentional processing.

#### Statistical Analyses

Statistical analyses followed the same general procedure as in Experiment 1. Internal consistency of the PSWQ was excellent (Cronbach’s $\alpha$ = 0.91). The average PSWQ score in this sample was *M* = 51.55 (*SD* = 9.01), closely matching the distribution observed in Experiment 1.

### Results

#### Questionnaire

As in Experiment 1, the effectiveness of the design manipulation was checked using the post-task questionnaire. Full details are reported in the Supplementary Material (Figure S4). The results confirmed that participants were strongly engaged in the gamified aspect of the task. The two threat-related hosts were rated more unpleasant than the neutral host, with no significant difference observed between the two threat conditions. The appearance of the threat-related hosts was rated as significantly more feared than that of the neutral host. Finally, participants reported they expected the hosts to appear throughout the task. These results support the effectiveness of the experiment.

#### Parity Judgment

Mean parity-judgment accuracy was high (*M* = 0.84, *SD* = 0.07). The best-fitting random-effects structure included random intercepts for participants and random slopes for trial number and digit position, both varying within participants (see Supplementary Material). The model explained a modest proportion of variance ($R^2_{\text {marginal}} = 0.01$, $R^2_{\text {conditional}} = 0.15$).

**Fig. 3:**
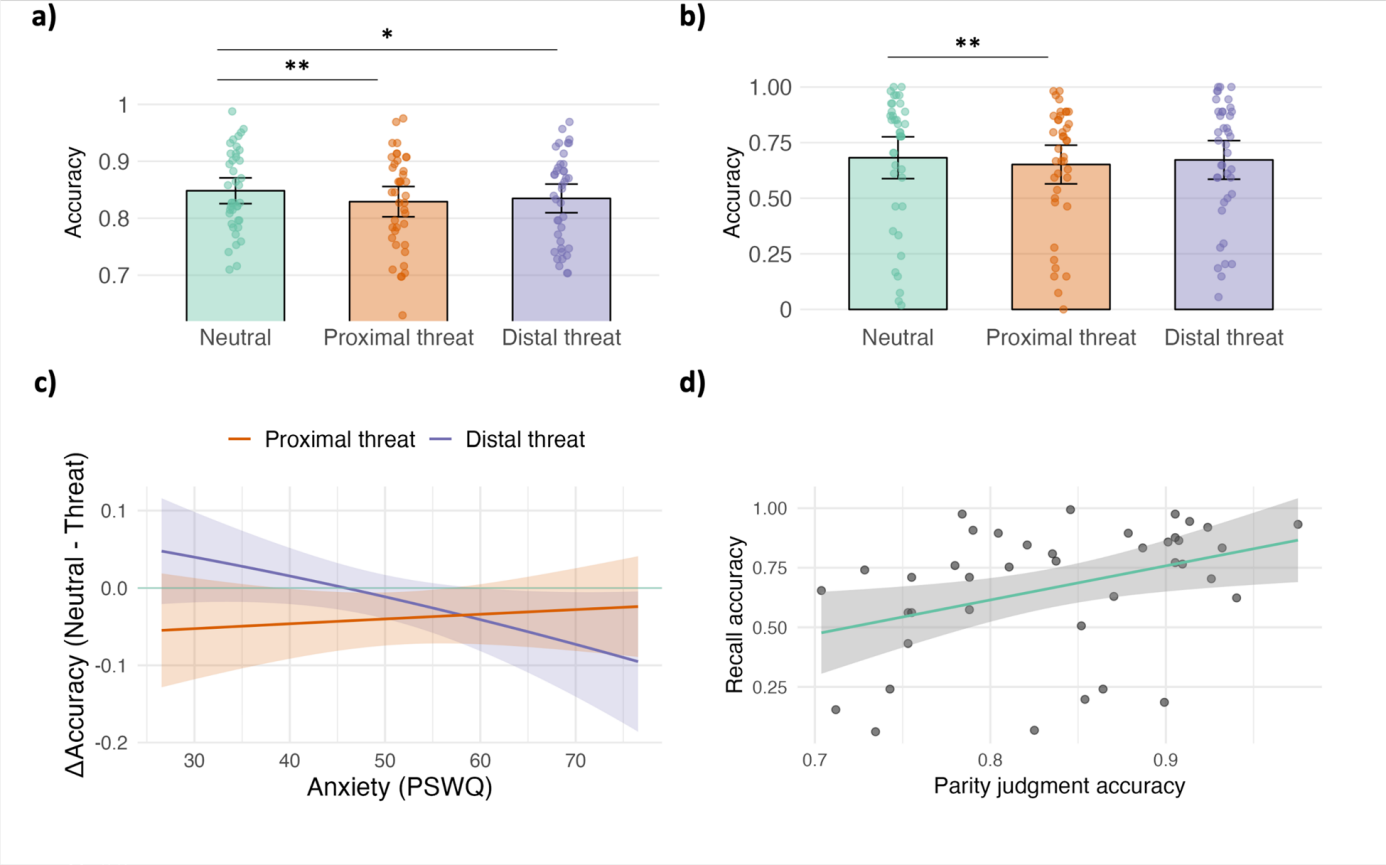
Illustration of accuracy patterns as a function of threat conditions and PSWQ scores in Experiment 2. **a**) Mean parity-judgment accuracy across threat conditions. **b**) Mean recall accuracy across threat conditions. **c**) Effect of PSWQ scores on the change in recall accuracy between threat and neutral conditions. **d**) Correlation between parity-judgment and recall accuracy. *Note.* For parity judgments, accuracy reflects the proportion of digits correctly classified. For recall, accuracy reflects the proportion of letters recalled in the correct serial position. In panels a) and b), vertical lines represent 95% confidence intervals; * $p < 0.05$, ** $p < 0.01$. In panel c), shaded areas represent 95% confidence intervals

Using this structure, removing Threat condition significantly worsened model fit, $\chi ^2(2) = 9.56$, $p = 0.008$, $\Delta \textrm{AICc} = 6$, indicating a significant main effect of Threat condition. Planned contrasts showed reduced accuracy relative to the Neutral condition in both the Proximal threat ($\beta = -0.15$, $\mathit{SE} = 0.05$, 95% CI [–0.24, –0.05], $z = -2.97$, $p = 0.003$) and Distal threat conditions ($\beta = -0.11$, $\mathit{SE} = 0.05$, 95% CI [–0.21, –0.01], $z = -2.17$, $p = 0.030$). Exploratory analyses confirmed that this threat effect was stable across task progression (see Supplementary material for details). The Threat condition main effect is shown in Fig. 3a.

Removing the Threat condition $\times$ PSWQ interaction did not worsen model fit, $\chi ^2(2) = 3.00$, $p = 0.22$, $\Delta \textrm{AICc} = -1$, indicating no evidence that anxiety modulated threat-related interference in this task.

#### Recall

Mean recall accuracy was moderate (*M* = 0.67, *SD* = 0.27). The best-fitting random structure included random intercepts for participants, and random slopes for trial number and letter position, both varying within participants (see Supplementary Material). The model explained an important proportion of variance ($R^2_{\text {marginal}} = 0.01$, $R^2_{\text {conditional}} = 0.52$).

Removing Threat condition significantly worsened fit, $\chi ^2(2) = 7.53$, $p = 0.02$, $\Delta \textrm{AICc} = 3.6$, indicating a significant main effect. Planned contrasts revealed lower recall accuracy in the Proximal threat condition than in the Neutral condition ($\beta = -0.22$, $\mathit{SE} = 0.08$, 95 % CI [–0.38, –0.06], $z = -2.69$, $p = 0.007$). In contrast, no difference in recall accuracy was found between the Distal threat condition and the Neutral condition ($\beta = -0.09$, $\mathit{SE} = 0.08$, 95 % CI [–0.25, 0.07], $z = -1.10$, $p = 0.27$). The Threat condition main effect is illustrated in Fig. 3b. An exploratory analysis revealed that the lower recall accuracy in the Proximal threat condition compared to the Neutral condition was mainly present in the first rather than the second part of the task (see Supplementary Materials for details).

The Threat condition $\times$ PSWQ interaction was significant, $\chi ^2(2) = 10.24$, $p = 0.006$, $\Delta \textrm{AICc} = 6.2$. Planned contrasts showed that the reduced recall accuracy in the Distal threat condition compared to the Neutral condition increases with PSWQ ($\beta = -0.20$, $\mathit{SE} = 0.08$, 95% CI [–0.36, –0.04], $z = -2.48$, $p = 0.01$), while no corresponding effect was found for the Proximal threat condition ($\beta = 0.04$, $\mathit{SE} = 0.08$, $p = 0.61$). The Threat condition x PSWQ interaction effect is illustrated in Fig. 3c. Parity-judgment and recall accuracy were positively correlated ($r = 0.38$, 95% CI [0.08, 0.62], $p = 0.01$), as shown in Fig. 3d.

Across both tasks, threat impaired performance, with convergent but task-specific effects. In the parity-judgment task, accuracy was high overall and showed a clear main effect of Threat condition. Both Proximal and Distal threat reduced accuracy relative to Neutral, with no evidence of modulation by cognitive anxiety. In the recall task, threat again impaired performance, but selectively. Recall was lower in the Proximal threat condition, whereas the Distal threat condition did not differ from Neutral on average. However, cognitive anxiety moderated threat-related interference, with recall accuracy in the Distal threat condition decreasing as PSWQ levels increased. Finally, parity-judgment and recall accuracy were positively correlated, indicating that participants who showed stronger threat-related disruption in attentional decisions also tended to exhibit greater impairment in WM maintenance.

## Pooled Analysis Across Experiments

### Rationale and Analytic Strategy

To formally assess the robustness and generalizability of the threat effects observed in Experiments 1 and 2, we combined the data from both studies into a single set of GLMMs including Experiment (1 vs. 2) as a between-subject factor. This pooled analysis allowed us to test whether the effects of Threat condition and its interaction with PSWQ differed across experiments, and to examine whether increasing task difficulty from Experiment 1 to Experiment 2 modulated these effects. For both tasks, we used the same outcome measures (parity-judgment and recall accuracy) and extended the fixed-effects structure to include Threat condition, PSWQ, Experiment (1 vs. 2), and their interactions. The random-effects structure was held identical to the best-fitting structures identified in the study-specific analyses for each task. It included random intercepts for participants as well as random slopes for item position (letter or digit, treated as a continuous predictor and z-scored within experiment) and trial number, both varying within participants.

### Pooled Parity Judgment

The full model explained a modest proportion of variance, $R^2_{\text {marginal}} = 0.01$, $R^2_{\text {conditional}} = 0.15$. Details about the random effect structure are reported in the Supplementary Material.

Removing Threat condition significantly worsened model fit, $\chi ^2(2) = 12.93$, $p = 0.001$, $\Delta \textrm{AICc} = 9$, indicating a significant main effect of Threat condition. The Threat condition $\times$ Experiment interaction was non-significant, $\chi ^2(2) = 0.87$, $p = 0.65$, $\Delta \textrm{AICc} = -4$, indicating no evidence that the impact of threat on parity-judgment accuracy differed between Experiments 1 and 2.

Planned contrasts showed reduced accuracy relative to the Neutral condition in both the Proximal threat ($\beta = -0.13$, $\mathit{SE} = 0.04$, 95% CI [-0.26, –0.01], $z = -3.10$, $p = 0.005$) and Distal threat conditions ($\beta = -0.14$, $\mathit{SE} = 0.04$, 95% CI [–0.31, –0.03], $z = -3.50$, $p = 0.003$).

Removing the Threat condition $\times$ PSWQ interaction worsened model fit, $\chi ^2(2) = 7.41$, $p = 0.02$, $\Delta \textrm{AICc} = 3$, indicating that anxiety modulated threat-related interference in this task. Removing Threat condition $\times$ PSWQ $\times$ Experiment interaction also worsened model fit, $\chi ^2(2) = 6.89$, $p = 0.03$, $\Delta \textrm{AICc} = 3$, suggesting that this modulation by anxiety differed between Experiments 1 and 2. Planned contrasts revealed that the Threat $\times$ PSWQ interaction took different forms across the two experiments.

In Experiment 1, accuracy in the Threat conditions increased relative to the Neutral condition as PSWQ scores rose. This pattern emerged marginally for the Proximal threat contrast ($\beta = 0.16$, *SE* = 0.07, 95% CI [0.02, 0.31], $z = 2.23$, $p = 0.065$) and significantly for the Distal threat contrast ($\beta = 0.25$, *SE* = 0.07, 95% CI [0.10, 0.39], $z = 3.32$, $p = 0.002$). In Experiment 2, by contrast, PSWQ did not modulate the threat–neutral difference for either the Proximal threat contrast ($\beta = 0.08$, *SE* = 0.05, 95% CI [–0.09, 0.26], $z = 1.59$, $p = 0.24$) or the Distal threat contrast ($\beta = 0.01$, *SE* = 0.05, 95% CI [–0.18, 0.23], $z = 0.22$, $p = 0.97$).

Direct comparison of these PSWQ slopes across experiments confirmed this divergence. The modulation of the Proximal threat contrast did not differ reliably between experiments ($\beta = 0.08$, *SE* = 0.09, 95% CI [–0.09, 0.26], $z = 0.92$, $p = 0.36$), whereas the modulation of the Distal threat contrast was significantly stronger in Experiment 1 than in Experiment 2 ($\beta = 0.23$, *SE* = 0.09, 95% CI [0.05, 0.41], $z = 2.58$, $p = 0.01$).

### Pooled Recall

The full model explained an important proportion of variance, $R^2_{\text {marginal}} = 0.04$, $R^2_{\text {conditional}} = 0.48$. Details about the random-effects structure are reported in the Supplementary Material.

Removing Threat condition significantly worsened model fit, $\chi ^2(2) = 14.64$, $p < .001$, $\Delta \textrm{AICc} = 10.7$, indicating a robust main effect of Threat condition on recall accuracy. By contrast, the Threat condition $\times$ Experiment interaction was non-significant, $\chi ^2(2) = 0.96$, $p = .62$, $\Delta \textrm{AICc} = -3$, providing no evidence that the magnitude of the threat-related recall impairment differed between Experiments 1 and 2. Planned contrasts confirmed reduced accuracy relative to the Neutral condition in both the Proximal threat condition ($\beta = -0.26$, *SE* = 0.07, 95% CI [–0.42, –0.10], $z = -3.89$, $p < .001$) and the Distal threat condition ($\beta = -0.17$, *SE* = 0.07, 95% CI [–0.33, –0.01], $z = -2.47$, $p < .001$).

Removing the Threat condition $\times$ PSWQ interaction also significantly worsened model fit, $\chi ^2(2) = 16.95$, $p < 0.001$, $\Delta \textrm{AICc} = 10.7$, indicating that cognitive anxiety modulated recall performance under threat. However, the Threat condition $\times$ PSWQ $\times$ Experiment interaction was non-significant, $\chi ^2(2) = 0.40$, $p = 0.82$, $\Delta \textrm{AICc} = -3.6$, suggesting that this modulation by anxiety did not differ between Experiments 1 and 2.

Planned contrasts showed that recall accuracy in the Distal threat condition decreased increasingly relative to the Neutral condition as PSWQ scores rose ($\beta = -0.18$, *SE* = 0.07, 95% CI [–0.35, –0.02], $z = -2.62$, $p = 0.02$), whereas PSWQ did not modulate the Proximal threat contrast ($\beta = 0.08$, *SE* = 0.07, 95% CI [–0.07, 0.25], $z = 1.24$, $p = 0.24$).

Across both tasks, the pooled analyses revealed coherent effects of anticipatory threat. Both proximal and distal threats reliably impaired accuracy in parity judgments and recall, with no evidence that these effects differed between Experiments 1 and 2. Cognitive anxiety showed task- and threat-specific influences. In parity judgments, accuracy in the threat conditions increased with PSWQ scores in Experiment 1, significantly for distal threat and marginally for proximal threat, indicating reduced threat-related attentional capture as anxiety increased under low task demands. This modulation was not observed in Experiment 2, and the distal threat effect was significantly smaller than in Experiment 1. For recall, accuracy decreased with increasing PSWQ only in the distal threat condition, and no evidence emerged suggesting that this effect varied across experiments. Together, these pooled results indicate that anticipatory threat captures attentional resources and impairs WM. They further show that anticipatory threat–related attentional interference diminishes as anxiety increases under low task complexity, while this modulation disappears when cognitive demands are high. Finally, WM impairment under distal threat tended to increase as anxiety levels rose.

## Discussion

The present study examined whether anticipatory threat, generated entirely in the absence of sensory stimuli, produces attentional capture and disrupts WM, and whether these effects vary with cognitive anxiety. Across two experiments and a pooled analysis, anticipating both proximal and distal threats reduced parity-judgment accuracy and recall performance, providing convergent evidence that anticipating threat captures attentional resources and impairs WM. These results align with contemporary accounts emphasizing the anticipatory nature of cognition (Friston et al., 2017; Hohwy, 2013) and emotion (Seth, 2013; Seth & Friston, 2016), showing that the expectation of an aversive event is cognitively costly even without perceptual cues.

This finding also fits appraisal theories, which place evaluative processes at the core of emotional elicitation (Grandjean et al., 2008; Sander, 2013; Scherer, 2009). Our design isolated the cognitive impact of anticipation itself, without exposing participants to threatening stimuli or engaging them in any mental imagery. In threat research, this pre-encounter phase is thought to mobilize resources to evaluate potential harms and prepare defensive actions (Mobbs et al., 2020). The present results provide a behavioral illustration of this mechanism. Indeed, both proximal and distal threats produced a reliable interference, suggesting that anticipating either immediate or temporally distant consequences is sufficient to tax attention and impair WM. This pattern is consistent with studies showing that presenting these same types of threat biases attention (Fournier & Koenig, 2024; Müller et al., 2016; Vogt et al., 2013; Wentura et al., 2014).

The impact of anticipatory threat on WM aligns with contemporary models. WM impairment has typically been observed when emotional stimuli are presented during the task (Chainay et al., 2023; Colliot et al., 2025; Garrison & Schmeichel, 2018; Plancher et al., 2019; Schweizer & Dalgleish, 2011, 2016). WM models further propose that internally generated representations also compete for limited attentional resources (Cowan, 2005), a prediction supported mainly in non-emotional contexts (Kiyonaga & Egner, 2016; Yu & Postle, 2021). The present results extend this principle to emotional contexts, showing that anticipatory threat creates attentional interference even without sensory input. The positive correlation between attentional interference and WM impairment across tasks is consistent with the TBRS model (Barrouillet et al., 2011; Plancher & Barrouillet, 2020), underscoring the central role of limited attentional resources in refreshing and maintenance.

Cognitive anxiety showed a more nuanced pattern. In Experiment 1, contrary to predictions, anticipatory threat-related attentional capture decreased as anxiety increased. This aligns with accounts suggesting that anxious individuals may engage in anxiety-regulation strategies when the task is manageable, allocating resources to the task to inhibit threat (Iacoviello et al., 2014; Naim et al., 2015; Zvielli et al., 2014). In Experiment 2, where task demands were higher, this compensatory pattern disappeared, consistent with evidence that anxiety-related failures of attentional control emerge under increased cognitive load (Angelidis et al., 2019; Shi et al., 2019). In contrast, WM impairment increased with anxiety in the pooled analysis, but only under distal threat. One possibility is that distal threat carries greater uncertainty than proximal threat. Its consequences are temporally delayed and ambiguously defined (e.g., how losing points would affect overall performance), and cognitive anxiety is especially sensitive to uncertainty (Milne et al., 2019). Differences in perceived intensity between the two threat types may also have contributed to this pattern.

### Limitations and Future Directions

Although both threat-related hosts were rated as significantly more unpleasant and fear-inducing than the neutral host, affective responses to such stimuli may nevertheless vary across individuals. For example, some participants may not perceive the screamer as aversive (e.g., individuals who enjoy horror-related stimuli), while the distal host may appear insufficiently threatening due to his friendly expression. Future studies could reduce this variability by using more standardized threat cues or by individually calibrating threat stimuli. A second limitation concerns the sustained credibility of the manipulation over time. Ratings of the credibility of the hosts’ potential appearance did not differ across task halves. Consistent with this, across the two tasks and two experiments, only one behavioral result showed a significant change between the first and second halves of the task. While these findings suggest overall stability of the manipulation, future designs could further reinforce credibility by introducing occasional filler-host appearances, thereby maintaining the expectation that any host could appear at any time. A third limitation relates to the modest effect sizes observed for the impact of anticipatory threat on WM. Although these effects were consistent across experiments, their small magnitude raises the possibility of methodological constraints or suggests an upper bound on the cognitive impact of threat anticipation in the absence of actual sensory threat input.

Despite these limitations, the paradigm provides a solid foundation for studying how anticipatory threat affects WM. Refining and replicating the design will help determine whether stronger or more credible cues yield larger effects, and whether anticipatory and perceptual threats exert comparable influences on WM.

The paradigm also enables more direct investigation of appraisal processes. A core implication of appraisal theories is that emotional responses vary depending on how events are evaluated with respect to concern relevance (Grandjean et al., 2008; Sander, 2013; Scherer, 2009). Continuous physiological measures (e.g., skin conductance), often treated as relevance markers (Schimmack, 2005), could provide time-resolved indices of anticipatory emotion.

Finally, adaptations could probe WM mechanisms more precisely. The current design does not dissociate attentional refreshing from verbal rehearsal mechanisms (i.e., silently repeating items). Future variants could introduce articulatory suppression to limit rehearsal and test whether anticipatory threat primarily disrupts refreshing, rehearsal, or both (Camos, 2015; Colliot et al., 2025).

## Supplementary Information

Below is the link to the electronic supplementary material.

Supplementary file 1 (pdf 1403 KB)

## Additional Information

### Availability of Data and Material

Data and material from the study are available through the Open Science Framework repository: https://osf.io/ucfj2/ .

### Code Availability

The preprocessing and analysis code is available via the Open Science Framework at: https://osf.io/ucfj2/ .

### Transparency and Openness

The level 2 requirement of the Transparency and Openness Guidelines (Nosek et al., 2015) was met for this article. The Journal Article Reporting Standards (Kazak, 2019) were followed. The sample size analysis, data exclusion, manipulation, and measurement were reported. Data, pre-treatment, code analyses, and material from the study are available through the Open Science Framework repository: https://osf.io/ucfj2/. Data were analyzed using R 4.3.1, within RStudio (version 2024.04). Models were fitted using the lme4 package version 1.1-35.2 (Bates et al., 2015), contrasts were performed using the emmeans package version 1.10.1 (Lenth, 2024).
