# Supplementary material for: When Threat Comes from Within: First Evidence that Anticipatory Emotion Impairs Working Memory
Source: Affect Sci. 2026 Mar 9;7(2):293–305. doi: 10.1007/s42761-026-00363-y (PMC13269615; doi:10.1007/s42761-026-00363-y)
Supplement: Supplementary file 1 — (pdf 1403 KB) [file 42761_2026_363_MOESM1_ESM.pdf]

When threat comes from within: first evidence that anticipatory emotion impairs working memory - Supplementary Material

Experiment 1

Method

Statistical analysis

To evaluate whether recall performance exhibited primacy and recency effects, we inspected accuracy as a function of letter position within each trial. As shown in Figure S1, performance was higher for letters presented at the beginning and at the end of the sequence, consistent with classical primacy–recency patterns. To account for this structure within the GLMMs, letter position was recoded according to the empirical ordering of performance (from lowest to highest accuracy), yielding the following sequence: 4, 3, 2, 5, 1. This data-driven recoding ensured that the random slope for item position captured meaningful variance associated with serial-position effects.

Results

Questionnaire

Regarding the desire to win, rated on a scale from 0 (not at all) to 1 (extremely), participants reported significantly higher scores than the neutral midpoint of 0.5 (M = 0.71, SD = 0.20; V = 743, $p < .001$), suggesting strong engagement with the gamified aspect of the task.

To assess the effectiveness of the anticipatory-threat induction materials, we examined whether the appearance of the threatening hosts was rated as more unpleasant than that of the neutral host. Ratings were collected on a continuous scale ranging from unpleasant (0), through neutral (0.5), to pleasant (1). Wilcoxon signed-rank tests were used due to violations of normality. The pleasantness rating of the neutral host (Sybille) did not differ significantly from the neutral midpoint (M = 0.58, SD = 0.22, V = 518.5, p = .15). In

Fig. S1 Recall accuracy as a function of letter position within the trial.

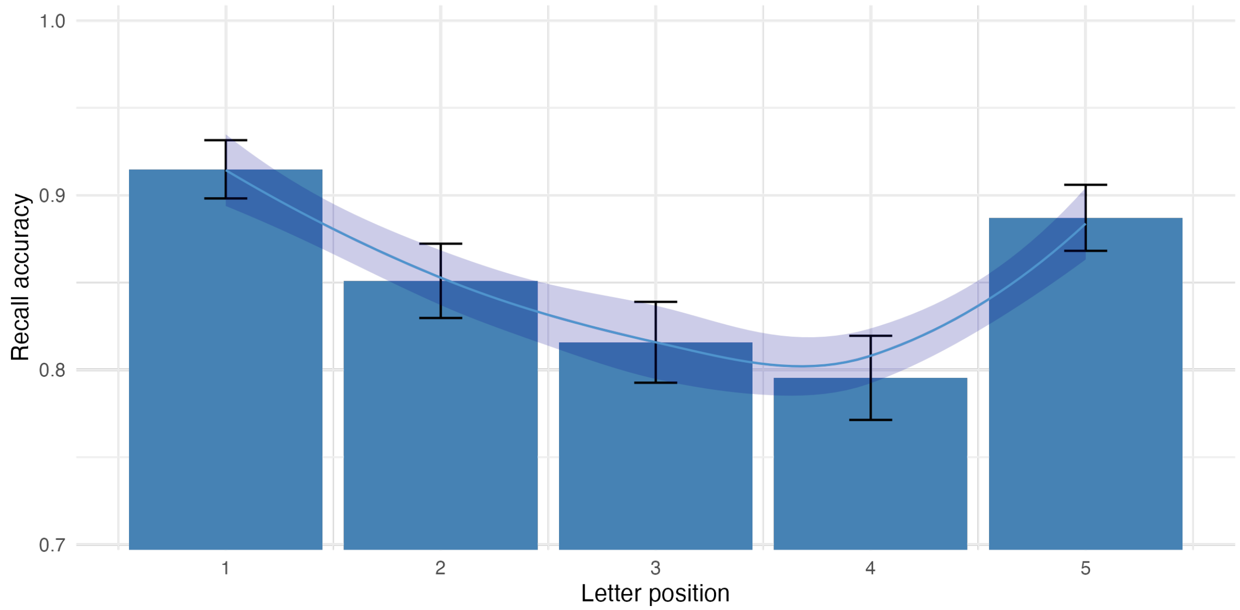

Note. Vertical lines represent 95% CI. The loess-smoothed trends are displayed with shaded areas indicating their 95% CI.

contrast, the proximal- and distal-threat hosts (Vlad and Max) were rated significantly below the neutral midpoint (proximal-threat host: $M = 0.24$, $SD = 0.20$, $V = 34$, $p < .001$; distal-threat host: $M = 0.23$, $SD = 0.19$, $V = 0$, $p < .001$). Moreover, both threat hosts were rated significantly more unpleasant than the neutral host (proximal-threat host vs. neutral host: $W = 9$, $p < .001$; distal-threat host vs. neutral host: $W = 41$, $p < .001$), whereas no difference in pleasantness ratings was observed between the two threat hosts ($W = 749.5$, $p = .63$). The distributions of pleasantness ratings for each host are presented in Figure S2A. This check ensured that the threat-related hosts were perceived as unpleasant, whereas the neutral host was evaluated as neutral.

We also examined whether participants feared the appearance of the hosts, using a continuous scale ranging from not at all feared (0) to extremely feared (1). Wilcoxon signed-rank tests revealed that the threat-associated hosts were rated as significantly more feared than the neutral host (neutral: $M = 0.13$, $SD = 0.20$; proximal threat: $M = 0.52$,

Fig. S2 Distribution of pleasantness and fear of appearance ratings for the three hosts as provided by participants in Experiment 1.

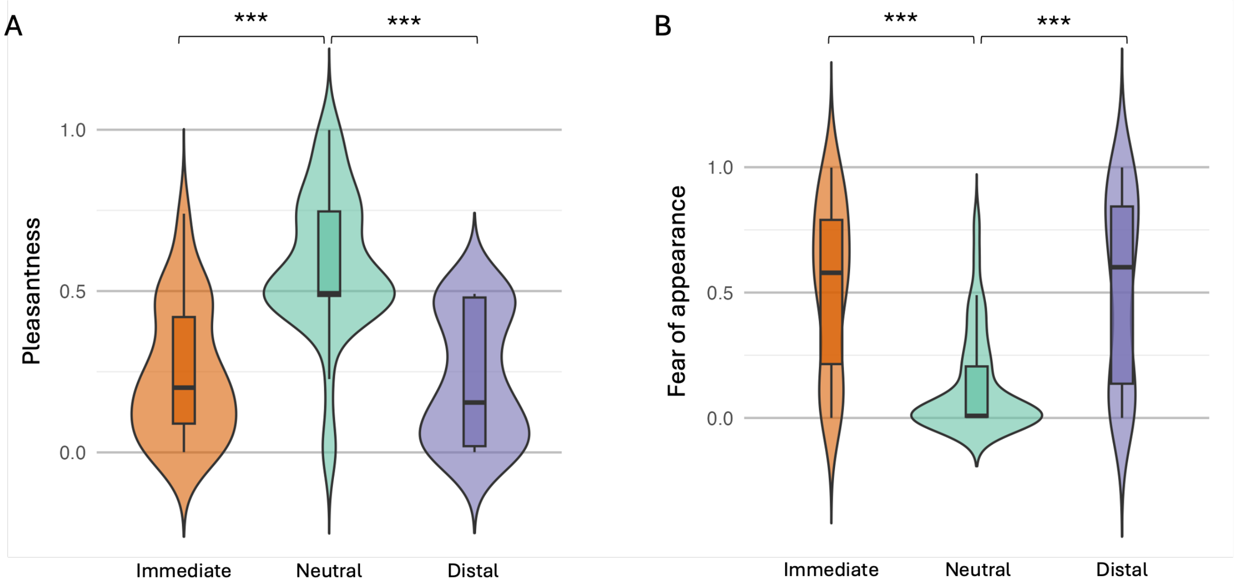

Note. Violin plots represent the distribution of ratings, and embedded boxplots indicate the median and interquartile range. (A) Pleasantness ratings ranged from 0 (unpleasant) to 1 (pleasant), with 0.5 indicating a neutral evaluation. (B) Fear of appearance ratings ranged from 0 (not at all feared) to 1 (extremely feared).

$SD = 0.33$; distal threat: $M = 0.52$, $SD = 0.37$; distal vs. neutral: $W = 755$, $p < .001$; proximal vs. neutral: $W = 746$, $p < .001$). In contrast, fear of appearance did not differ between the two threat-related hosts ($W = 817.5$, $p = .87$). Results are illustrated in Figure S2B. These findings show that, as expected, the two threat-related hosts were perceived as more fear-inducing than the neutral host, confirming the effectiveness of the anticipatory-threat induction.

To assess task credibility, we tested whether participants believed the hosts would appear during both parts of the task. Participants were asked to indicate their expectations regarding the appearance of a host before and after the mid-task break, using a continuous scale from not at all (0) to extremely (1). Results indicated that participants moderately

expected a host to appear, with ratings significantly above the first quartile of the scale (0.25) both before the break ($M = 0.45$, $SD = 0.27$, $V = 6105$, $p < 0.001$) and after the break ($M = 0.47$, $SD = 0.31$, $V = 5966.5$, $p < 0.001$). These findings suggest that participants maintained a consistent expectation of a host appearance throughout the task.

Parity judgment

Regarding the identification of random effects in the GLMM fitted on parity-judgment accuracy, the maximal structure justified by the design was first fitted. It included random intercepts and random slopes for all within-subject predictors (Threat condition, trial number, and digit position). This structure was specified as:

$$(1 + \text{Threat condition} + \text{Trial number} + \text{Digit position} \,|\, \text{Participant}).$$

All possible random-effects substructures, ranging from the maximal model to a model including only a random intercept for participants, were tested. The results of this analysis are reported in Table S1. The following structure yielded the lowest AICc:

$$(1 + \text{Threat condition} + \text{Trial number} + \text{Digit position} \,|\, \text{Participant}).$$

However, it exhibited very high correlations among random slopes ($\rho > 0.90$), indicating potential over-parameterization. To address this issue, we removed correlations among random effects and refitted the model using an uncorrelated structure:

$$(1 + \text{Threat condition} + \text{Trial number} + \text{Digit position} \,||\, \text{Participant}).$$

This uncorrelated structure yielded the best-fitting specification. The retained structure included random intercepts for participants (SD = 0.65), as well as random slopes for trial number (SD = 0.28) and digit position (SD = 0.29).
The adjusted intraclass correlation coefficient (ICC = 0.11) indicated that approximately 11% of the variance in parity-judgment accuracy was attributable to stable between-participant differences.

Table S1

Model comparison for identifying the best-fitting random-effects structure for parity-judgment accuracy

Random structure	df	AICc	ΔAICc
Trial number + Digit position || Participant	9	8544.93	0.00
Trial number | Participant	9	8588.22	43.30
Digit position | Participant	9	8592.04	47.12
Participant	7	8635.36	90.44
Threat condition | Participant	12	8637.61	92.69
Trial number + Digit position | Participant*	12	8526.85	-18.07
Threat condition + Trial number + Digit position | Participant	21	–	–
Threat condition + Digit position | Participant	16	–	–
Threat condition + Trial number | Participant	16	–	–

Notes. Model evaluation was performed using the full fixed-effects structure (main and interaction effects between *Threat condition* and *PSWQ*). * Although this model yielded a lower AICc, its random-slope correlations exceeded 0.9, indicating potential over-parameterization. "|" indicates random slopes correlated with the random intercept; "||" indicates random slopes specified without correlations. Models for which AICc and ΔAICc are not reported failed to converge and were therefore not considered further.

The main analysis was complemented with an exploratory analysis aimed at testing whether the effect of Threat condition on parity-judgment accuracy varied over time. This analysis was performed on the model used to test our hypotheses, including main and interaction effects between Threat condition and PSWQ, and the best-fitting random-effects structure. A Partition variable was created by dividing Trial number into two blocks (a first block of 14 trials and a last block of 13 trials), and added to the model along with its interactions with PSWQ.

The comparison between a model including Partition (and its interactions with PSWQ)

and a model additionally including the Threat condition × Partition interaction indicated that adding this interaction did not improve model fit, $\chi^2(2) = 0.21$, $p = 0.90$, $\Delta\text{AICc} = -3.8$. This result suggests that the influence of threat on parity-judgment accuracy remained stable across the task.

Recall

With regard to identifying random effects in the GLMM fitted on recall accuracy, the maximal structure justified by the design was first fitted. It included random intercepts and random slopes for all within-subject predictors (Threat condition, trial number, and letter position). This structure was specified as:

$$(1 + \text{Threat condition} + \text{Trial number} + \text{Letter position} \,|\, \text{Participant}).$$

All possible random-effects substructures, ranging from the maximal model to a model including only a random intercept for participants, were tested. The results of this analysis are reported in Table S2. The following structure yielded the lowest AICc:

$$(1 + \text{Threat condition} + \text{Trial number} + \text{Letter position} \,|\, \text{Participant}).$$

However, this model showed very high random-slope correlations ($\rho > 0.80$), indicating over-parameterization.

Consequently, correlations among random effects were removed, and the model was refitted using an uncorrelated structure, which yielded the final best-fitting specification:

$$(1 + \text{Threat condition} + \text{Trial number} + \text{Letter position} \,||\, \text{Participant}).$$

The retained structure included random intercepts for participants (SD = 1.12), and uncorrelated random slopes for trial number (SD = 0.41) and letter position (SD = 0.41).

The adjusted intraclass correlation coefficient (ICC = 0.28) indicated that 28% of the variance in recall accuracy was attributable to stable between-participant differences.

An exploratory analysis was conducted to assess whether the effect of Threat condition on recall accuracy varied over time. Trial number was divided into two partitions (a first block

Table S2

Model comparison for identifying the best-fitting random-effects structure for recall accuracy

Random structure	df	AICc	ΔAICc
Trial number + Letter position || Participant	9	3858.61	0.00
Letter position | Participant	9	3881.97	23.35
Trial number | Participant	9	3914.16	55.54
Participant	7	3954.39	95.78
Trial number + Letter position | Participant*	12	–	–
Threat condition | Participant	12	–	–
Threat condition + Trial number + Letter position | Participant	21	–	–
Threat condition + Trial number | Participant	16	–	–
Threat condition + Letter position | Participant	16	–	–

Notes. Model evaluation was performed using the full fixed-effects structure (main and interaction effects between *Threat condition* and *PSWQ*). * Although this model yielded a lower AICc, its random-slope correlations exceeded 0.80, indicating potential over-parameterization. "|" indicates correlated random slopes; "||" indicates uncorrelated random slopes. Models for which AICc and ΔAICc are not reported failed to converge and were therefore not considered further.

of 14 trials and a last block of 13 trials). The Partition factor was added to the model using the same random-effects structure as in the main analysis, together with its interactions with Threat condition and PSWQ.

The comparison between a model including Partition (and its interactions with PSWQ) and a model additionally including the Threat condition × Partition interaction indicated that adding this interaction did not improve model fit, $\chi^2(2) = 1.18$, $p = 0.55$, $\Delta\text{AICc} = -2.8$. This result suggests that the influence of threat on recall accuracy remained stable across the task.

Experiment 2

Method

Statistical analysis

To evaluate whether recall performance exhibited primacy and recency effects, we inspected accuracy as a function of letter position within each trial. As shown in Figure S3, performance was higher for letters presented at the beginning and at the end of the sequence, consistent with classical primacy–recency patterns. To account for this structure within the GLMMs, letter position was recoded according to the empirical ordering of performance (from lowest to highest accuracy), yielding the following sequence: 5, 4, 3, 6, 2, 1. This data-driven recoding ensured that the random slope for item position captured meaningful variance associated with serial-position effects.

Fig. S3 Recall accuracy as a function of letter position within the trial.

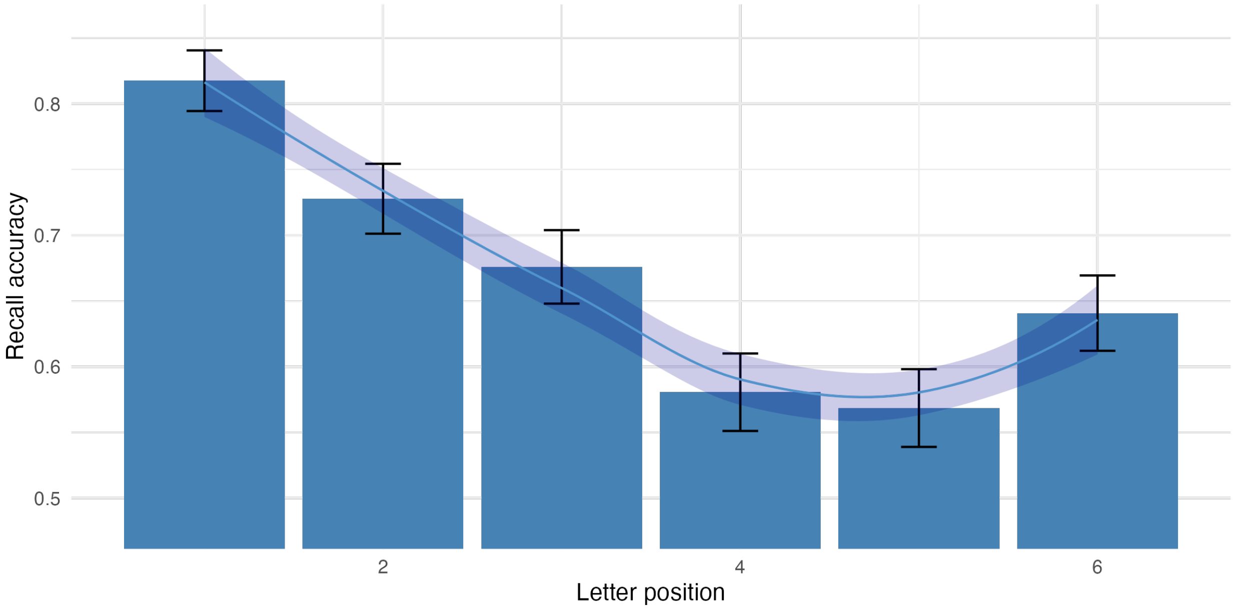

Note. Vertical lines represent 95% CI. The loess-smoothed trends are displayed with shaded areas indicating their 95% CI.

Results

Questionnaire

As in Experiment 1, participants in Experiment 2 reported a strong desire to win, rated on a scale from 0 (not at all) to 1 (extremely). Their scores were significantly higher than the neutral midpoint of 0.5 ($M = 0.74$, $SD = 0.20$; $V = 770$, $p < .001$), indicating robust engagement with the gamified aspect of the task.

To replicate the manipulation check of Experiment 1, we examined whether the threatening hosts were rated as more unpleasant than the neutral host. Ratings were collected on a continuous scale ranging from unpleasant (0), through neutral (0.5), to pleasant (1). Wilcoxon signed-rank tests were used due to violations of normality. The pleasantness rating of the neutral host did not differ significantly from the neutral midpoint ($M = 0.56$, $SD = 0.25$, $V = 479$, $p = .36$). In contrast, the proximal- and distal-threat hosts were rated significantly below the neutral midpoint (proximal-threat host: $M = 0.24$, $SD = 0.22$, $V = 40$, $p < .001$; distal-threat host: $M = 0.19$, $SD = 0.21$, $V = 18$, $p < .001$). Moreover, both threat-related hosts were rated significantly more unpleasant than the neutral host (proximal-threat host vs. neutral host: $W = 59$, $p < .001$; distal-threat host vs. neutral host: $W = 39.5$, $p < .001$), whereas no difference in pleasantness ratings was observed between the two threat-related hosts ($W = 679$, $p = .25$). Results are illustrated in Figure S4A. These findings replicate those of Experiment 1 and confirm that the threat-related hosts were perceived as more unpleasant than the neutral host.

We also examined whether participants feared the appearance of the hosts, using a continuous scale ranging from not at all feared (0) to extremely feared (1). Fear of appearance ratings differed between the hosts. The neutral host received low fear ratings ($M = 0.11$, $SD = 0.19$). In contrast, both the proximal-threat host ($M = 0.46$, $SD = 0.36$) and the distal-threat host ($M = 0.53$, $SD = 0.36$) were rated significantly higher in fear than the neutral host (proximal-threat host vs. neutral host: $W = 680$, $p < .001$; distal-threat host vs. neutral host: $W = 777$, $p < .001$). No significant difference in fear of

Fig. S4 Distribution of pleasantness and fear of appearance ratings for the three hosts as provided by participants in Experiment 2.

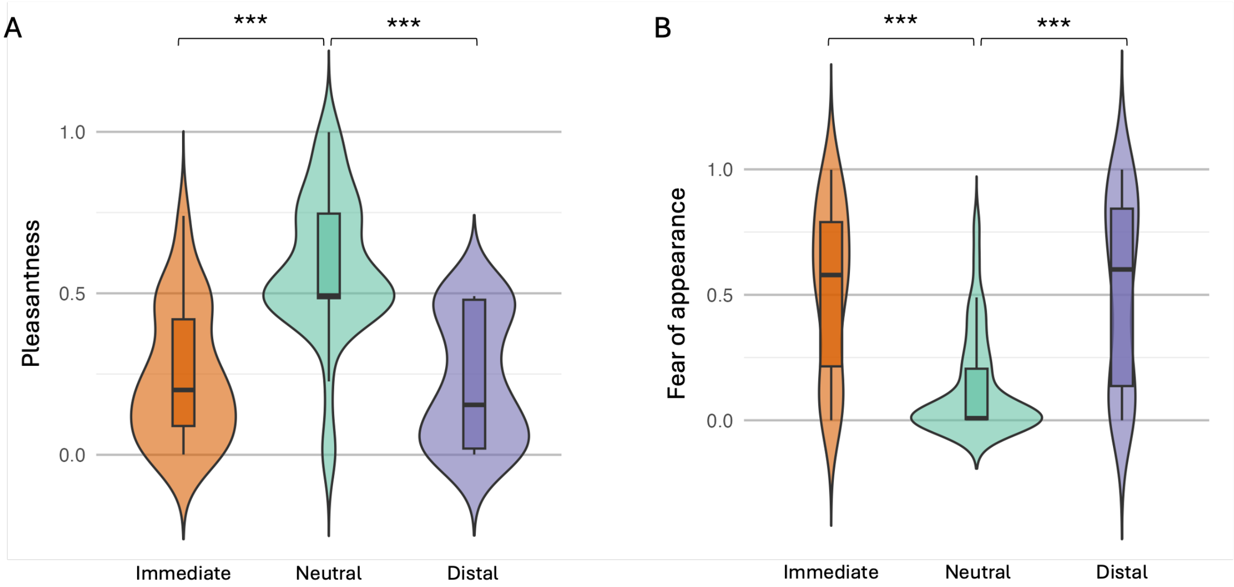

Note. Violin plots represent the distribution of ratings, and embedded boxplots indicate the median and interquartile range. (A) Pleasantness ratings ranged from 0 (unpleasant) to 1 (pleasant), with 0.5 indicating a neutral evaluation. (B) Fear of appearance ratings ranged from 0 (not at all feared) to 1 (extremely feared).

appearance was observed between the two threat-related hosts ($W = 927$, $p = .22$). Results are illustrated in Figure S4B. These findings replicate those of Experiment 1 and confirm that the threat-related hosts were perceived as more fear-inducing than the neutral host.

To assess task credibility, we examined whether participants believed the hosts would appear during both parts of the task. Participants indicated their expectations regarding a host's appearance before and after the mid-task break, using a continuous scale from not at all (0) to extremely (1). Ratings were significantly above the first quartile of the scale (0.25) both before the break ($M = 0.46$, $SD = 0.27$, $V = 6222$, $p < 0.001$) and after the break ($M = 0.48$, $SD = 0.30$, $V = 6114$, $p < 0.001$), suggesting that participants maintained a consistent expectation of a host appearance throughout the task.

Parity judgment

Regarding the identification of random effects in the GLMM fitted on parity-judgment accuracy, the maximal structure justified by the design was first fitted. It included random intercepts and random slopes for all within-subject predictors (Threat condition, trial number, and digit position). This structure was specified as:

$$(1 + \text{Threat condition} + \text{Trial number} + \text{Digit position} \,|\, \text{Participant}).$$

All possible random-effects substructures, ranging from the maximal model to a model including only a random intercept for participants, were then compared. The results of this analysis are reported in Table S3. The structure yielding the lowest AICc was:

$$(1 + \text{Trial number} + \text{Digit position} \,|\, \text{Participant}).$$

The retained structure included random intercepts for participants (SD = 0.59), as well as random slopes for trial number (SD = 0.20) and digit position (SD = 0.41). Random-slope correlations were modest in magnitude: the correlation between the participant intercept and the trial-number slope was $\rho = -0.15$, between the intercept and the digit-position slope $\rho = -0.23$, and between the trial-number and digit-position slopes $\rho = 0.30$.
An exploratory analysis evaluated whether the effect of Threat condition varied as a function of task progression. Trial number was divided into two blocks (first 14 trials vs. last 13 trials), forming a Partition factor that was added to the model together with its interactions with PSWQ. Comparing this model with an extended version including the Threat condition × Partition interaction revealed no evidence for temporal modulation, $\chi^2(2) = 3.95$, $p = 0.14$, $\Delta\text{AICc} = 0$. This indicates that the influence of threat on parity-judgment accuracy remained stable across the task.

Table S3

Model comparison for identifying the best-fitting random-effects structure for parity-judgment accuracy (Experiment 2)

Random structure	df	AICc	ΔAICc
Trial number + Digit position | Participant	12	16269.22	0.00
Digit position | Participant	9	16317.05	47.83
Trial number | Participant	9	16606.02	336.80
Participant	7	16651.27	382.05
Threat condition | Participant	12	16655.11	385.88
Threat condition + Trial number + Digit position | Participant	21	–	–
Threat condition + Digit position | Participant	16	–	–
Threat condition + Trial number | Participant	16	–	–

Notes. Model evaluation was performed using the full fixed-effects structure (main and interaction effects between *Threat condition* and *PSWQ*). "|" indicates random slopes correlated with the random intercept. Models for which AICc and ΔAICc are not reported failed to converge and were therefore not considered further.

Recall

With regard to identifying random effects in the GLMM fitted on recall accuracy, the maximal structure justified by the design was first fitted. It included random intercepts and random slopes for all within-subject predictors (Threat condition, trial number, and letter position):

$$(1 + \text{Threat condition} + \text{Trial number} + \text{Digit position} \,|\, \text{Participant}).$$

All possible random-effects substructures, ranging from this maximal model to a model including only a random intercept for participants, were tested. The full results are

presented in Table S4. The lowest AICc was obtained for:

$$(1 + \text{Trial number} + \text{Digit position} \,|\, \text{Participant}).$$

The retained structure included random intercepts for participants (SD = 1.73), and correlated random slopes for trial number (SD = 0.34, $\rho = 0.30$) and letter position (SD = 0.69, ρ with intercept = 0.25; ρ with trial number = 0.33). The adjusted intraclass correlation coefficient (ICC = 0.48) indicated that nearly half of the variance in recall accuracy reflected stable between-participant differences.

Table S4

Model comparison for identifying the best-fitting random-effects structure for recall accuracy (Experiment 2)

Random structure	df	AICc	ΔAICc
Trial number + Letter position | Participant	12	5811.64	0.00
Letter position | Participant	9	5858.27	46.63
Threat condition + Trial number | Participant	16	6146.40	334.76
Trial number | Participant	9	6155.88	344.24
Threat condition | Participant	12	6186.21	374.58
Participant	7	–	–
Threat condition + Trial number + Letter position | Participant	21	–	–
Threat condition + Letter position | Participant	16	–	–

Notes. Model evaluation was performed using the full fixed-effects structure (main and interaction effects between *Threat condition* and *PSWQ*). Models for which AICc and ΔAICc are not reported failed to converge and were therefore excluded. "|" indicates correlated random slopes.

The main analysis was complemented by an exploratory test assessing whether the effect of Threat condition on recall accuracy varied across the task. Trial number was divided into two blocks (first 14 trials vs. last 13 trials), forming a Partition factor. This variable was

added to the model with the same random-effects structure used in the main analysis, together with its interactions with PSWQ and Threat condition.

Adding the Threat condition × Partition interaction significantly improved model fit, $\chi^2(2) = 10.49$, $p = 0.005$, ΔAICc $= 6.50$, indicating that the influence of threat on recall accuracy varied over time.

Follow-up contrasts showed that this effect was driven by a significantly smaller neutral–proximal threat difference in the second partition than in the first ($\beta = 0.53$, $z = 3.18$, $p = 0.001$). In contrast, the neutral–distal threat difference did not significantly vary across partitions ($\beta = 0.16$, $z = 0.95$, $p = 0.34$).

Pooled analysis across experiments

Pooled parity judgment

The random structure included in the GLMM fitted on parity-judgment accuracy was:

$$(1 + \text{Trial number} + \text{Digit position} \mid \text{Participant}).$$

This structure yielded random intercepts for participants (SD = 0.62), together with random slopes for trial number (SD = 0.24) and digit position (SD = 0.38). Random-slope correlations were as follows: the correlation between the participant intercept and the trial-number slope was $\rho = 0.06$, between the intercept and the digit-position slope $\rho = 0.22$, and between the trial-number and digit-position slopes $\rho = 0.49$. The adjusted Intraclass Correlation Coefficient (ICC = 0.10) indicated that 10% of the variance in accuracy was attributable to stable between-participant differences.

Pooled recall

The random structure included in the GLMM fitted on recall accuracy was:

$$(1 + \text{Trial number} + \text{Letter position} \mid \text{Participant}).$$

This structure yielded random intercepts for participants (SD = 1.55), together with random slopes for trial number (SD = 0.37) and digit position (SD = 0.57). Random-slope

correlations were as follows: the correlation between the participant intercept and the trial-number slope was $\rho = 0.05$, between the intercept and the digit-position slope $\rho = 0.53$, and between the trial-number and digit-position slopes $\rho = 0.30$. The adjusted Intraclass Correlation Coefficient (ICC = 0.42) indicated that 42% of the variance in accuracy was attributable to stable between-participant differences.
